# The provision of psychological care for patients with mesothelioma and their caregivers: an Italian survey

**DOI:** 10.3389/fpubh.2026.1879469

**Published:** 2026-08-05

**Authors:** Antonella Granieri, Isabella Giulia Franzoi, Maria Domenica Sauta, Francesca Barbagli, Carolina Mensi, Alessandro Marinaccio, Michela Bonafede

**Affiliations:** 1 Department of Psychology, University of Turin, Turin, Italy; 2 Centro Operativo Regionale (COR) Lombardy, Occupational Health Unit, Fondazione Institute for Research, Hospitalization and Healthcare (IRCCS) Ca' Granda, Ospedale Maggiore Policlinico of Milan, Milan, Italy; 3 Occupational and Environmental Medicine, Epidemiology and Hygiene Department, Italian Workers' Compensation Authority (INAIL), Rome, Italy

**Keywords:** asbestos, cancer, mesothelioma, oncology, pleura, psychological distress, psychological intervention, survey

## Abstract

**Background:**

Mesothelioma is a rare, aggressive tumor that profoundly affects patients and caregivers. However, few studies address psychological interventions for this population, either nationally or internationally. This study therefore aims to map the current state of psychological care provided by the Regional Operating Centers. (CORs) of the Italian National Mesothelioma Registry and other health and welfare facilities in Italy.

**Methods:**

The survey lasted approximately 15 min.

**Results:**

The final sample includes 15 CORs and 57 other facilities. Among them, 66.7% (*n* = 10) of CORs and 5.3% (*n* = 3) of other facilities lack a psychologist. Only one COR includes a psychologist at diagnosis, compared to 12.9% of other facilities. Psychological interventions are offered by only 25% of all centers. Where psychologists are present, 100% of respondents consider them useful; in their absence, 45.5% of CORs and all other facilities consider their inclusion valuable. Interventions for mesothelioma patients and families are provided in two of four CORs and in 37 of 52 other facilities with a psychologist. In seven facilities, interventions target only patients, and in three, they are specific to this population of patients and caregivers.

**Conclusions:**

Although national and international guidelines emphasize the importance of interdisciplinary care for mesothelioma patients and their families, many still lack adequate support. Closing this gap is urgent, as most healthcare professionals recognize the value of such integration—both when they experience it and when it is absent in their workplace.

## 1 Introduction

Internationally, environmental and occupational asbestos exposure remains an active and pressing problem, as it is currently estimated that asbestos is one of the leading causes of work-related death. More than half are due to oncological diseases, particularly to mesothelioma (1, 2), a rare and aggressive form of cancer whose major known cause is asbestos exposure (3, 4).

Asbestos was widely produced and used in Italy, particularly between the 1960s and 1980s, before its extraction, processing, marketing and use were banned in 1992. Given the long latency period between first exposure and the manifestation of mesothelioma, asbestos remains a public health issue that is still relevant today. In particular, the Italian National Mesothelioma Registry (Registro Nazionale Mesoteliomi—ReNaM) of the Italian Workers' Compensation Authority (Istituto Nazionale per l'Assicurazione contro gli Infortuni sul Lavoro—INAIL) was established in 2002 as a national epidemiological surveillance system based on Operative Regional Centers (Centri Operativi Regionali-CORs). The latest data reported 37,003 cases of mesothelioma diagnosed between 1993 and 2021, with an average age at diagnosis of 71 years (5).

Both national and international literature show that a diagnosis of mesothelioma has a major impact on patients and their caregivers (6). In particular, it can lead to anxiety and depression (7, 8), an impoverished emotional life (9), somatization (1) and a deterioration in their quality of life (10). In addition, there is a need among patients and caregivers for more information about mesothelioma, its progression and the associated care tasks (11, 12).

Psychological support plays a crucial role in helping both patients and caregivers cope with the emotional burden, uncertainty, and social isolation often associated with mesothelioma. The presence of trained mental health professionals can improve treatment adherence, enhance communication with healthcare staff, and reduce emotional distress. Despite this, psychological care is often underrepresented in oncology settings, and few structured programs exist specifically for mesothelioma patients in Italy.

Previous research on the psychological impact of mesothelioma on patients and their caregivers, as well as on the psychological interventions provided (13, 14) highlights both the importance of integrating psychological interventions into the management of patients and caregivers and the lack of published studies on the topic. This is a major concern for public health and patient advocacy and raises the question of whether this gap is due to an actual lack of experience in the interdisciplinary care of mesothelioma patients and their caregivers, or to clinical practice that struggles to produce outcome and process studies or scientific publications on the subject.

Thus, the aim of this study was therefore to map the clinical psychology services that patients with mesothelioma and their families could find in terms of diagnosis and intervention in the Italian territory, both in CORs and in other health and welfare facilities.

In particular, our objective was to explore the use of tools to assess the psychological distress and psychosocial needs of mesothelioma patients and their families, as well as the presence and organizational characteristics of active psychological support services aimed at this population.

The specific aims of this research are investigate the integration of psychology in the care of mesothelioma patients and their caregivers in Italy, exploring whether a psychologist is available, the involvement of the psychologist in the communication of the diagnosis; the use of specific tools to assess the psychological distress and psychosocial needs of these patients and their families; the provision and characteristics of psychological interventions provided, and to assess the perceived usefulness of the presence of a psychologist for other health professionals.

## 2 Methods

### 2.1 Survey instrument

We developed an *ad hoc* semi-structured interview as a digital survey using Google Forms, targeting healthcare professionals (psychologists, physicians, nurses, department heads, etc.) involved in CORs and other health and welfare facilities. Four authors (AG, IGF, MDS, and FB) defined the areas to be explored in the interview, the specific questions for each area, and the sequence of questions. Two authors (MB and CM), from different institutions and with different roles in caring for patients and family members, then revised the items.

The final version of the survey included up to 54 questions: 44 closed-response or multiple-choice questions and 10 open-ended questions. The survey incorporated skip logic so that, based on responses to key questions (e.g., presence of a psychologist, use of assessment tools, provision of interventions), some subsequent questions were automatically activated or excluded. This approach allowed the survey path to be tailored to each facility, reducing perceived length and increasing the relevance of the data collected.

The questions were organized into eight topics, each focusing on a specific aspect of the integration of psychology in interdisciplinary care (Table 1).

**Table 1:** Structure of the survey.

1. General facility and respondent information	2. Presence of a psychologist	3. Diagnosis communication	4. Psychological distress and needs evaluation	5. Psychological interventions	6. Psychological interventions characteristics	7. Perceived usefulness	8. Outcome evaluation
Geographic region	Current presence of a psychologist	Whether the activity is provided in the facility	Whether the activity is provided in the facility	Whether the activity is provided in the facility	Intervention setting (individual, couple, family, group)	Perceived utility of the psychologist (when present or not)	Outcome/process studies of the interventions offered
Facility type (hospital, COR, association, etc.)	How long has the psychologist been present	Presence of a psychologist during diagnosis communication	Tools used	Population involved (patients, caregivers, both)	Intervention typology (psychological consultation, short/long term therapy)	Perceived utility of the psychologist in the diagnosis communication	
Respondent professional role	Past presence of a psychologist		Population involved (patients, caregivers, both)		Involved professional figures	Perceived utility of the assessment tools	
					Provision of different interventions for patients and caregivers	Perceived utility of the psychological interventions	
					Provision of specific interventions for mesothelioma patients and caregivers compared to standard psychological interventions for all cancer patients and caregivers		
**Objective**	**Objective**	**Objective**	**Objective**	**Objective**	**Objective**	**Objective**	**Objective**
Mapping the geographical distribution of facilities and their types	Investigating the integration of psychology into care	Investigating whether psychology is integrated into the communication of diagnoses in facilities that provide such diagnoses	Exploring psychological assessment and the use of assessment tools in facilities	Exploring the provision of psychological interventions	Exploring the characteristics of provided psychological interventions	Analyzing the perceived usefulness of psychological assessments and interventions	Researching whether and how psychological interventions are monitored

The survey was not self-administered, data were collected through researcher-administered telephone interviews, during which a member of the research team asked the questions and entered participants' responses into the electronic survey form.

### 2.2 Survey administration

The list of CORs and their contacts was retrieved via INAIL. The other facilities were selected from a preliminary list of reference healthcare facilities for each region and from a list of active psycho-oncology centers in the national territory provided by the Italian Society of Psycho-Oncology (Società Italiana di Psico-Oncologia—SIPO). For each area, a list of potential participants was compiled: a total of 341 facilities were identified, including 20 CORs (5.87%) and 320 other facilities (93.84%).

Each facility was subsequently contacted and invited to participate in the study, primarily by telephone and, less frequently, by email (in which case a follow-up phone appointment was scheduled). Since the study involved healthy subjects, participation was voluntary and no sensitive data on respondents were collected, Ethics Committee involvement was not required. A description of the study was provided to each respondent by telephone, and verbal informed consent was obtained during the interview. Consent was then recorded on the interview form. The data collected were processed in full compliance with applicable privacy and sensitive data regulations.

### 2.3 Participants

Data were collected from July 2022 to December 2024. All 20 Italian CORs active at the time of enrollment were contacted (the COR of the Campania region was inactive at the time of the survey). Five CORs (25%) chose not to respond to the survey: four CORs did not respond to telephone calls or emails, one COR requested more information about the survey but chose not to respond, and one COR was unavailable for the survey at the agreed time. Of the 320 other facilities contacted, 57 (17.8%) agreed to participate. Thus, our final sample consisted of 72 facilities (21.11%) out of the 341 contacted: 15 CORs (20.83%) and 57 other facilities (79.17%).

The interview lasted approximately 15 min.

### 2.4 Statistical analyses

The frequency analysis was conducted with the Statistical Package for the Social Sciences (SPSS; IBM Corp., Armonk, NY, USA), 30th version.

## 3 Results

**Figure 1:**
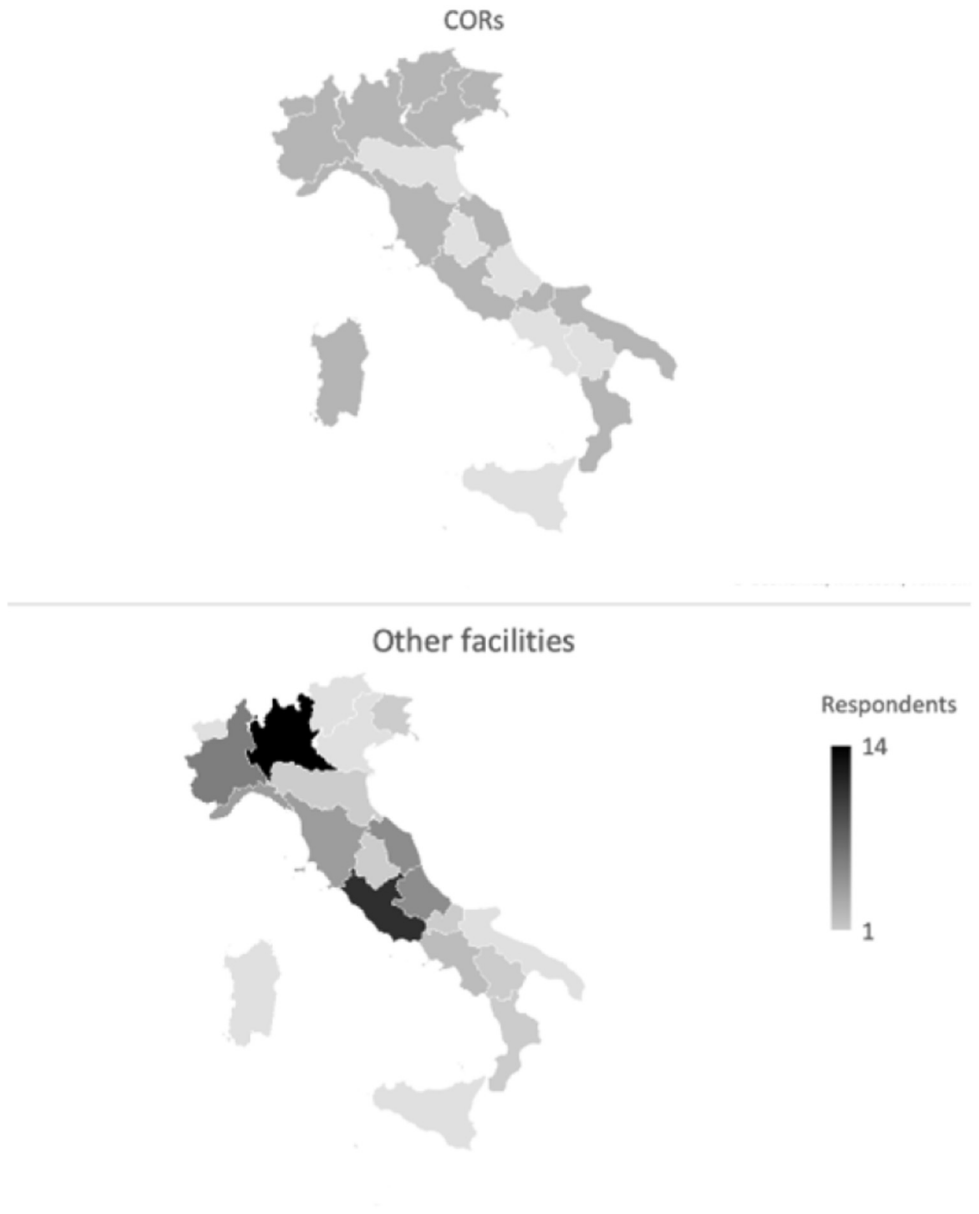
Geographical distribution of respondents.

For what concerns the distribution of respondents on the national territory, the 15 CORs that agreed to participate were from Aosta Valley, Apulia, Autonomous Province of Bolzano and Autonomous Province of Trento (both in the Trentino Alto Adige region), Calabria, Friuli Venezia Giulia, Lazio, Liguria, Lombardy, Marche, Molise, Piedmont, Sardinia, Tuscany, and Veneto (Figure 1).

Among the other facilities, 14 were from Lombardy (24.6%); 11 from Lazio (19.3%); six from Piedmont (10.5%); five from Marche (8.8%), five from Abruzzo (8.8%); four from Liguria (7%); four from Tuscany (7%); two from Campania (3.5%); one (1.8%) from respectively Basilicata, Calabria, Emilia-Romagna, Friuli Venezia Giulia, Molise and Umbria (Figure 1).

The other results of the survey are summarized in Table 2.

**Table 2:** Results of the survey.

Variables	CORs (*n* = 15)	Other facilities (*n* = 57)
1. General facility and respondent information		
Facility type (hospital, COR, association, etc.)	*NA*	• Hospitals: 35 (61.4%) • Associations: 11 (19.3%) • Territorial services: 6 (10.5%) • Scientific recovery and care institutes: 3 (5.3%) • Hospices: 2 (3.5%)
2. Presence of a psychologist		
Current presence of a psychologist	• Yes: 4 (26.6%) • No: 10 (66.6%) • Do not know: 1 (6.6%)	• Yes: 52 (91.2%) • No: 3 (5.3%) • Do not know: 2 (3.5%)
How long has the psychologist been present	*In the CORs with a psychologist (n = 4)* • Since 2021: 1 (25%) • Since a few months: 1 (25%) • Do not know: 2 (50%)	*In the facilities with a psychologist (n = 52)* • More than 10 years: 32 (61.5%) • 5–10 years: 6 (11.5%) • 1–5 years: 3 (5.8%)
Past presence of a psychologist	*In the CORs without a psychologist/not knowing about the presence of a psychologist (n = 11)* • No: 9 (81.8%) • Do not know: 2 (18.2%)	*In the facilities without a psychologist/not knowing about the presence of a psychologist (n = 5)* • Yes: 2 (40%) • No: 1 (20%) • Do not know: 2 (40%)
3. Diagnosis communication		
Whether the activity is provided in the facility	*In the CORs with a psychologist (n = 4)* • Yes: 1 (25%) • No: 3 (75%)	*In the facilities with a psychologist (n = 52)* • Yes: 31 (59.6%) • No: 17 (32.7%) • Do not know: 4 (7.7%)
Presence of a psychologist during diagnosis communication	*In the CORs providing the diagnosis communication (n = 1)* • Yes: 1 (100%)	*In the facilities providing the diagnosis communication (n = 31)* • Yes: 4 (12.9%) • No: 26 (83.9%) • Do not know: 1 (3.2%)
4. Psychological distress and needs assessment		
Whether the activity is provided in the facility	*In the CORs with a psychologist (n = 4)* • Yes: 2 (50%) • No: 2 (50%)	*In the facilities with a psychologist (n = 52)* • Yes: 27 (51.9%) • No: 12 (23.1%) • Do not know: 4 (7.7%) • Not answered: 9 (17.3%)
Population involved (patients, caregivers, both)	*In the CORs using assessment tools (n = 2)* • Both patients and caregivers: 2 (100%)	*In the facilities using assessment tools (n = 27)* • Both patients and caregivers: 7 (25.9%) • Patients only: 18 (66.7%) • Do not know: 2 (7.4%)
5. Psychological interventions		
Whether the activity is provided in the facility	*In the CORs with a psychologist (n = 4)* • Yes: 2 (50%) • No: 2 (50%)	*In the facilities with a psychologist (n = 52)* • Yes: 37 (71.1%) • No: 5 (9.6%) • Do not know: 1 (1.9%) • Not answered: 9 (17.3%)
Population involved (patients, caregivers, both)	*In the CORs providing psychological interventions (n = 2)* • Both patients and caregivers: 2 (100%)	*In the facilities providing psychological interventions (n = 37)* • Both patients and caregivers: 29 (78.4%) • Only patients: 7 (18.9%) • Do not know: 1 (2.7%)
Intervention setting (individual, couple, family, group)	*In the CORs providing psychological interventions (n = 2)* • One-to-one setting: 2 (100%)	*In the facilities providing psychological interventions (n = 37)* • One-to-one setting: 19 (51.4%) • One-to-one/family/group setting: 7 (18.9%) • One-to-one/family/group/couples setting: 5 (13.5%) • According to needs: 4 (10.8%) • Do not know: 2 (5.4%)
Intervention typology (psychological consultation, short/long term therapy)	*In the CORs providing psychological interventions (n = 2)* • Brief psychotherapy: 1 (50%) • According to needs: 1 (50%)	*In the facilities providing psychological interventions (n = 37)* • Psychological counseling: 6 (16.2%) • Psychological support: 11 (29.7%) • Brief psychotherapy: 1 (2.7%) • According to needs: 15 (40.5%) • Do not know: 4 (10.8%)
Professionals involved	*In the CORs providing psychological interventions (n = 2)* • Psychologists: 1 (50%) • Psychotherapists: 1 (50%)	*In the facilities providing psychological interventions (n = 37)* • Psychotherapists: 26 (70.3%) • Psychologists and other healthcare professionals: 11 (29.7%)
Provision of different interventions for patients and caregivers	*In the CORs providing psychological interventions (n = 2)* • Yes: 1 (50%) • Do not know: 1 (50%)	*In the facilities providing psychological interventions (n = 37)* • Yes: 31 (83.8%) • No: 3 (8.1%) • Do not know: 3 (8.1%)
Provision of specific interventions for mesothelioma patients and caregivers vs. provision of standard psychological interventions for all cancer patients and caregivers	*NA (CORs are only involved in the care of mesothelioma patients and caregivers)*	*In the facilities providing psychological interventions (n = 37)* • Yes: 3 (8.1%) • No: 27 (72.9%) • Do not know: 7 (18.9%)
Number of patients/caregivers to whom interventions have been provided	*In the CORs providing psychological interventions (n = 2)* • 30 to 80 patients • 100 caregivers	*ND according to the responses given*
7. Perceived usefulness		
Perceived utility of the psychologist (whether the psychologist is present or not)	*In the CORs with a psychologist (n = 4)* • Yes: 4 (100%) *In the CORs without a psychologist/not knowing about the presence of a psychologist (n = 11)* • Yes: 5 (45.5%) • No: 6 (54.5%) • Do not know: 2 (18.2%)	*In the facilities with a psychologist (n = 52)* • Yes: 52 (100%) *In the facilities without a psychologist/not knowing about the presence of a psychologist (n = 5)* • Yes: 3 (60%) • Do not answered: 2 (40%)
Perceived utility of the psychologist in the diagnosis communication	*In the CORs providing the diagnosis communication with a psychologist (n = 1)* • Yes: 1 (100%)	*In the facilities providing the diagnosis communication with a psychologist (n = 4)* • Yes: 3 (75%) • No: 1 (45%)
Perceived utility of the assessment tools	*In the CORs using assessment tools (n = 2)* • Yes: 1 (50%) • No: 1 (50%)	*In the facilities using assessment tools (n = 27)* • Yes: 27 (100%)
Perceived utility of the psychological interventions	*In the CORs providing psychological interventions (n = 2)* • Yes: 2 (100%)	*In the facilities providing psychological interventions (n = 37)* • Yes: 36 (97.3%) • Do not know: 1 (0.7%)
8. Outcome evaluation		
Outcome/process studies of offered interventions	*In the CORs providing psychological interventions (n = 2)* • Yes: 1 (50%) • No: 1 (50%)	*In the facilities providing psychological interventions (n = 37)* • Yes: 19 (51.3%) • No: 8 (21.6%) • Do not know: 10 (27%)

Among other facilities, most of the respondents (61.4%) belonged to Hospitals (public hospitals, accredited private clinics, hospital societies and territorial healthcare and welfare societies). Other respondents were Associations (19.3%), territorial services (e.g., outpatient clinics, counseling centers, local socio-medical facilities; 10.5%), scientific recovery and care institutes (5.3%) and hospices (3.5%).

Psychologists are present in only 26.6% of CORs, compared to 91.2% of other facilities.

Psychological interventions are provided in 50% of CORs and 71.1% of other facilities with a psychologist. In other facilities, the intervention targets only patients in 18.9% of cases. Finally, 100% of both CORs and other facilities with a psychologist consider their presence useful, while in facilities without a psychologist, 45.5% of CORs and 60% of other facilities consider it useful.

## 4 Discussion

The importance of interdisciplinary care, as well as the significant role of mental health professionals in promoting mental health and wellbeing, is now widely recognized (15). In this context, we examined the presence and characteristics of psychological interventions for patients with mesothelioma and their caregivers, which is crucial since both patients and caregivers may experience a range of psychological symptoms (12, 16, 17).

When surveying various facilities that care for patients with mesothelioma and their families about whether a psychologist is part of their healthcare team, we found that fewer than thirty percent of CORs surveyed reported having a psychologist on their team. In the two CORs where a psychologist is present, this has only been the case recently. In contrast, more than ninety percent of the other surveyed facilities (public hospitals, licensed private hospitals, hospital societies, territorial healthcare and welfare societies, associations, territorial services, scientific recovery and care institutes) have a psychologist on their team, and in most cases for a longer period—more than 10 years in over half of the cases.

These results are not surprising, as CORs are not responsible for patient care, while most other facilities are primarily responsible for it. The presence of psychological staff even in the CORs is a positive indication of the increasing integration of psychology into the activities of ReNaM, supported by various BRIC projects (INAIL Research Plan 2016–2018 P9O1 BRIC ID 59, Research Plan 2019–2021 P9O1 BRIC ID55, and Research Plan 2022–2024 P9O1–BRIC ID66) that include psychology in the interdisciplinary team. Unfortunately, we are also aware that the presence of a psychologist in most CORs is funded by such projects, and at their conclusion, CORs may lose their ability to sustain the financial expense of a psychologist (we already know this has occurred in a COR). In other facilities, the presence of at least one psychologist in their team in most cases is certainly a positive sign. However, the present survey does not allow us to map the amount of available psychological staff in relation to the number of users, nor the specific training they have in relation to mesothelioma, which would certainly be interesting to assess in further studies.

This pattern is consistent with reports from other countries, where access to psycho-oncology services for patients with rare cancers remains highly heterogeneous and is often dependent on local organizational resources rather than being systematically integrated into multidisciplinary care pathways (18, 19). These findings suggest that organizational models promoting the routine inclusion of psychologists within multidisciplinary teams could improve equity of access to supportive care.

Regarding the communication of the diagnosis, it is well known that this can be an overwhelming experience, leading to both psychological and relational consequences, especially in the case of a mesothelioma diagnosis (15, 20, 21). Research highlights the importance of warmer, more respectful and participatory communication when delivering the diagnosis (20, 22). When a psychologist is present during the communication of the diagnosis, patients and their families can receive answers to questions about the clinical aspects of the disease and its physical impact, but also an initial containing space for the somatopsychic aspects that accompany the experience of the illness. Even if direct interaction with the clinical psychologist is limited, actively avoided, or if it is not possible to discuss the various clinical-psychological options, knowing that a mental health professional is part of the team, getting to know this professional, and being aware that this professional works closely with the oncologist increases the likelihood of accessing psychological treatment protocols even at a later stage. Nonetheless, this occurs in only a few cases, as our survey also shows. Communication of the diagnosis is not a primary COR activity, so it is not surprising that it is performed in only one COR, and that in this COR, which has a psychologist on its team, the psychologist is present during the communication. However, in the other facilities with a psychologist on their team, communication of the diagnosis is usually an elective activity, but a mental health professional is rarely present. In our opinion, this may be due to the low number of psychologists relative to the number of users. Unfortunately, facilities that not only emphasize the importance of having a psychologist present at the time of diagnosis but also have enough mental health staff to make this possible are rare and exemplary. The importance of promoting greater integration of psychologists in this activity is supported by respondents' positive opinions on its usefulness, even though the sample size is small.

International recommendations consistently emphasize that the communication of difficult diagnoses should occur within a multidisciplinary framework, with timely access to psychosocial support whenever appropriate. Early involvement of mental health professionals has been associated with improved emotional adjustment, better communication with healthcare providers, and greater patient satisfaction (18, 23).

Although there is international consensus on the important role that psychological distress, psychological needs, and other psychosocial factors play in the disease experience of both patients and caregivers, assessment tools are used in only half of CORs and other facilities where a psychologist is present. Moreover, these tools are mostly considered useful in other facilities, while CORs are not always positive about them. In addition, in many facilities psychological assessment tools are directed only at patients, excluding caregivers. These data confirm the value of tools created specifically to assess the psychological distress and needs of mesothelioma patients and their family members (24, 25), and highlight the importance of raising awareness among COR staff regarding the value of psychological assessment tools. Indeed, in CORs, this may sometimes be undervalued, as it could be seen as an additional burden for patients and their relatives—who also undergo an interview in CORs exploring domestic, occupational and family asbestos exposure—rather than as a way of meeting their needs.

A proper assessment of the psychological distress and needs of patients and caregivers is highly relevant because it can lead to tailored psychological care (6, 13, 14, 26). It is encouraging that among the four CORs with a psychologist, two offer psychological interventions, and such interventions are provided in 71.1% of other facilities. However, while CORs specialize in mesothelioma, in other facilities that care for all oncology patients, interventions specifically developed for mesothelioma patients and their caregivers are offered in only 8.1% of facilities. Additionally, respondents could not indicate the number of mesothelioma patients and their caregivers who have received care in their facilities. We are concerned that in some cases, very few interventions for mesothelioma patients and their caregivers may have been offered over time. The need for extensive integration of psychology into care protocols and for effective psychological support often conflicts with limitations imposed by National Health Systems, which frequently provide an insufficient number of sessions, sometimes limited to a single session (27, 28). Consequently, families affected by cancer, especially those facing socioeconomic disadvantages, often lack adequate support. For mesothelioma, this is exacerbated by its rarity, resulting in different facilities treating only a few patients over several years and mental health professionals not always having specific training. Psychological interventions, both in CORs and other facilities, include psychological counseling, psychological support, and short-term or long-term psychotherapy, and may vary according to the specific needs of patients and caregivers. The professionals responsible for these interventions are most often psychologists and psychotherapists, or mental health professionals working with other healthcare providers. However, we cannot collect information on the actual involvement of mesothelioma patients and caregivers in these activities or on the specific training of the professionals involved. Internationally, several guidelines highlight the need to offer specialized psychological support to mesothelioma patients and caregivers (13), but often do not provide details on the required professional skills for delivering psychological support to this population. The need for caregiver support is also frequently overlooked. In our sample, in 18.9% of other facilities, interventions are provided only to patients. Recognizing the specific needs of this oncology population enables the testing of new integrated care protocols and the development of policy approaches to implement more effective services while respecting the unique characteristics of users and planned budgets (29, 30).

The outcome or process of psychological interventions is evaluated in approximately half of CORs and other facilities. However, a recent systematic review of the literature revealed a lack of evaluation of intervention effectiveness (13). In some facilities, interventions may be offered to mesothelioma patients and caregivers and evaluated, but without specific publications on these efforts. More likely, facilities do evaluate the psychological interventions they offer, but none are specific to mesothelioma. As a result, mesothelioma patients represent only a small percentage of the samples of oncological patients and caregivers included in outcome or process studies of these interventions, making it difficult to determine whether such interventions are truly useful for this group.

Finally, we emphasize that 100% of both CORs and other facilities with a psychologist consider their presence useful, while 45.5% of CORs and 60% of other facilities without a psychologist consider it useful to add a psychologist to their team. These findings reinforce the growing recognition of the value of integrating psychology into interdisciplinary care and highlight the need to work with other healthcare professionals to increase the perceived value of psychological work in health contexts whenever patients and caregivers are involved.

Overall, our findings are consistent with the growing international recognition that psychosocial care should be considered an essential component of high-quality cancer care rather than an optional supportive service. Strengthening organizational policies that facilitate the integration of psychological professionals into routine mesothelioma care may contribute to more patient-centered and equitable healthcare delivery (31).

## 5 Clinical implications

Patients with mesothelioma and their caregivers express a clear need for psychological support due to the particularly challenging disease they or a family member are facing (8, 13, 15). The presence of a psychologist in healthcare facilities caring for patients with mesothelioma can be highly beneficial, since an authentically interdisciplinary team present from the time of diagnosis can help patients and caregivers process the diagnosis and cope with feelings of shame, guilt, isolation, anger, or grief that often accompany the diagnosis (14, 32). However, our survey shows that these professionals are not always present among healthcare staff, and psychological care is often not specific to this group, which may affect the overall care provided. There is a need for healthcare policies aimed at standardizing the provision of specialized care nationwide.

## 6 Limitations

This study has some limitations concerning the generalizability of the results. First, the cross-sectional design and reliance on self-report data are notable limitations. No instruments were used to assess the construct validity of the items, and no external experts were consulted to define the set of items. Additionally, the use of telephone interviews and digital forms may have introduced response biases, such as social desirability and acquiescence bias, or differences in response rates across regions and facility types. Finally, given the descriptive nature of the study and the limited number of participating facilities, the findings may not be fully generalizable to all Italian healthcare settings. Future studies should include patient or caregiver perspectives to deepen understanding of unmet psychological needs.

An additional limitation of this study is the potential for selection bias, as participation was voluntary and healthcare facilities with a greater interest in psychological care may have been more likely to respond. Furthermore, the descriptive cross-sectional design of the survey does not allow causal inferences or assessment of changes over time.

## 7 Conclusions

Psychological interventions are still not always offered to patients with mesothelioma and their caregivers, but when a psychologist is present, their usefulness is recognized by other healthcare professionals. Psychological interventions can help patients and caregivers move beyond the diagnosis and its traumatic effects, reaching a psychological state where they can find vitality—even when facing their own death or the loss of a loved one—thanks to a restored ability to tolerate, symbolize, mentalize, and narrate the traumatic and vital aspects of the disease without denial (13, 32).

Our findings highlight the need for national health policies that formally recognize the psychological dimension of mesothelioma care, promoting the inclusion of psychologists in multidisciplinary teams and supporting their specialized training. Furthermore, standardized psycho-oncological protocols should be developed to ensure equitable access to psychological services across Italian regions.

## Data Availability

The raw data supporting the conclusions of this article will be made available by the authors, without undue reservation.
